# Transthyretin is a novel innate immune effector against Gram negative bacteria

**DOI:** 10.1371/journal.ppat.1014086

**Published:** 2026-03-23

**Authors:** Tania Bernabé, María Verd, Guillem Ramis, Alex González-Alsina, Mohammad Qadi, Margalida Mateu-Borrás, Antonio Doménech-Sánchez, Sebastián Albertí

**Affiliations:** 1 Instituto Universitario de Investigación en Ciencias de la Salud (IUNICS) Universidad de las Islas Baleares and Instituto de Investigación Sanitaria de les Illes Balears (IDISBA), Palma de Mallorca, Spain; 2 Servicios Científico-técnicos. Universidad de las Islas Baleares, Palma de Mallorca, Spain; The University of Melbourne Faculty of Medicine Dentistry and Health Sciences, AUSTRALIA

## Abstract

*Pseudomonas aeruginosa* is an opportunistic pathogen that frequently causes severe bloodstream and respiratory infections, yet the interactions between this bacterium and the innate immune system remain poorly investigated. In this study we identified transthyretin, the transporter protein of thyroid hormone and retinol, as a novel binding partner of the bacterium. We show that transthyretin binds to lipopolysaccharide via lipid A. Transthyretin binding induces the agglutination of transthyretin-bacteria complexes and a reduction in bacterial viability. Mapping studies reveal that the N-terminal region of transthyretin mediates bacterial interaction, and a synthetic peptide derived from this domain exhibits potent bactericidal activity against a broad collection of *P. aeruginosa* isolates as well as other Gram-negative bacteria by disrupting membrane integrity. These findings identify transthyretin as an endogenous antimicrobial factor and uncover cryptic antimicrobial activity within its N-terminal region. Beyond extending the functional repertoire of transthyretin, these results suggest a novel role for this protein in innate defense.

## Author summary

In this study, we identify human transthyretin (TTR)—best known as the carrier protein for thyroid hormone and retinol-binding protein—as a previously unrecognized binding partner of *Pseudomonas aeruginosa*. We show that TTR recognizes the lipid A portion of the bacterial lipopolysaccharide (LPS), a highly conserved feature of Gram-negative pathogens. Functionally, TTR binding promotes bacterial agglutination and reduces viability. We further map the bactericidal activity of TTR to its N-terminal region. A synthetic peptide derived from this region (TTR1′) directly disrupts bacterial membrane integrity and displays potent antimicrobial activity against a broad panel of Gram-negative clinical isolates, markedly reducing viability in most strains tested.

These findings expand the functional repertoire of TTR beyond its classical role as a carrier protein. The discovery that TTR—and particularly its N-terminal fragment—possesses direct antimicrobial properties reveals a new facet of innate immunity and suggests that host amyloidogenic proteins may act as natural antimicrobial agents.

## Introduction

The innate immune response to microbial pathogens involves a rapid and coordinated activation of multiple host defense mechanisms. Critical components of this response include pathogen recognition, induction of inflammation, and phagocytosis—processes that are essential for controlling and clearing infections. The bloodstream represents a particularly important compartment for studying innate immunity, as it contains a diverse array of soluble immune effectors that play key roles in limiting systemic dissemination of pathogens.

*Pseudomonas aeruginosa* is a major cause of morbidity and mortality in hospitalized patients with nosocomial pneumonia or bloodstream infections [1,2] and frequently co-infects or causes secondary infections in patients with COVID-19 [3,4]. Additionally, *P. aeruginosa* is the leading cause of chronic, life-threatening pulmonary infections in individuals with cystic fibrosis [5]. The clinical burden of *P. aeruginosa* infection is exacerbated by its intrinsic and acquired resistance to a broad spectrum of antibiotics, complicating treatment and contributing to poor patient outcomes [6].

The clinical course of *P. aeruginosa* infection is influenced by multiple factors, including bacterial virulence, antimicrobial resistance, and the effectiveness of host immune responses. Notably, a substantial proportion of *P. aeruginosa*-related deaths occur within the first 24–72 hours of infection [7,8], underscoring a potential failure of early innate immune defenses. Although the importance of innate immunity in controlling *P. aeruginosa* is well recognized, additional studies are needed to identify novel innate host defense factors.

In this study we applied an unbiased proteomic analysis aimed at identifying previously unrecognized host serum proteins that interact with *P. aeruginosa*, we discovered a novel interaction between *P. aeruginosa* and transthyretin (TTR).

TTR is a homotetrameric soluble protein (55 kDa) that is synthesized in both the liver and the choroid plexus and circulates in blood (3–7 μM) and in the cerebrospinal fluid (CSF) (0·1–0·4 μM) [9]. Its normal physiological role is to serve as a carrier for thyroxine and retinol-binding protein [9,10]. Destabilization of the TTR tetramer can initiate amyloidosis, a rare disorder in which misfolded protein aggregates accumulate and disrupt the function of specific organs. However, TTR has not previously implicated in interactions with bacterial pathogens. This unexpected finding led us to further characterize the TTR–*P. aeruginosa* interaction and investigate its functional significance in the context of host-pathogen dynamics.

## Results

### *P. aeruginosa* binds human transthyretin

To characterize the earliest interactions between the microorganism and innate immune components present in host serum, we performed a global proteomic analysis of human serum proteins that associate with the bacterial surface during initial contact. A pooled human serum sample, supplemented with EDTA to prevent complement activation, was incubated with the *P. aeruginosa* reference strain PAO1. After incubation, bacteria were subjected to extensive washing, and surface-associated proteins were subsequently eluted. Proteins recovered from both the final wash and the elution fraction were digested and identified by mass spectrometry. To exclude proteins appearing in the eluted fraction due to high serum abundance rather than true bacterial association, we considered only those proteins whose relative abundance in the elution fraction—averaged across five independent experiments—was at least twofold higher than in the final wash.

Twenty-two proteins identified by mass spectrometry were found to interact with *P. aeruginosa* (Table 1). Table S1 provides a detailed overview of the identified proteins and proteomic analysis parameters. Immunoglobulins were excluded from the analysis because they represent an adaptive, antigen-specific immune response rather than the innate immune response, which was the focus of this study. Twenty-one of the proteins we identified have been previously implicated in the pathogenesis of *P. aeruginosa* or other bacterial pathogens (references in Table 1), although direct binding to bacteria has not been demonstrated for all. In addition, we detected transthyretin (TTR), a host protein that, to our knowledge, has not been previously reported to interact with bacterial pathogens or to play a direct role in bacterial pathogenesis.

**Table 1:** Human serum proteins bound to *P. aeruginosa* PAO1.

Protein	Reference (a)
Apolipoprotein A IV	[11]
Apolipoprotein B100	[12]
C4b-binding protein	[13]
CD5 antigen-like	[14]
Ceruloplasmin	[15]
Clusterin	[16]
Coagulation factor V	[17]
Complement C1 component	[18]
Complement factor H	[19]
Complement factor H-related protein 1	[20]
Fibrinogen	[21]
Ficolin-2	[18]
Haptoglobin	[22]
Histidine-rich glycoprotein	[23]
Inter-alpha-trypsin-inhibitor	[24]
Kininogen-1	[25]
Myeloperoxidase	[26]
Plasminogen	[19]
Prothrombin	[27]
Serum amyloid A	[28]
Transthyretin	–
Vitronectin	[16]

a) References reporting the roles of these proteins in the pathogenesis of bacterial infections.

TTR is a homotetrameric soluble protein that is synthesized in both the liver and the choroid plexus and circulates in blood and in the CSF. Its primary physiological role involves the transport of the hormone thyroxine and retinol-binding protein [9,10]. However, the role of TTR in the interactions with bacterial pathogens remains completely unknown.

**Fig 1:**
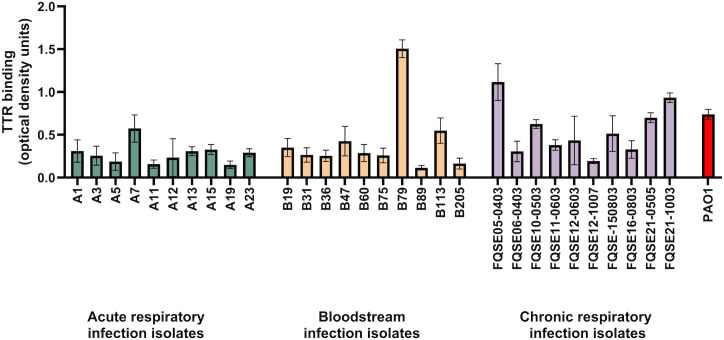
Binding of transthyretin to *P. aeruginosa* clinical isolates. The binding of TTR to *P. aeruginosa* clinical isolates and the reference strain PAO1, incubated in 10% heat-inactivated human serum, was analyzed by whole-cell ELISA. Bound TTR was detected with a specific polyclonal antibody. Optical densities from negative control wells incubated with PBS instead of human serum were always below 0.1 and were subtracted from the experimental values. Data are representative of three independent experiments performed in duplicate; errors bars indicate standard deviation.

To validate the interaction of TTR with *P. aeruginosa* beyond its binding to the reference strain PAO1, we evaluated the binding of TTR to a set of twenty clinical isolates. Microtiter plate wells coated with bacterial cells of the clinical isolates and the reference strain PAO1 were incubated in 10% heat-inactivated human serum and after extensive washing, bound TTR was detected with a specific antibody (Fig 1). All tested isolates bound TTR suggesting that the binding of TTR may be a common feature of *P. aeruginosa* clinical isolates.

### Identification of the TTR binding surface component in *P. aeruginosa*

**Fig 2:**
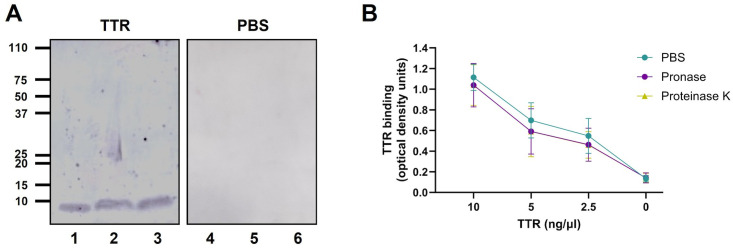
Identification of the transthyretin binding site in *P. aeruginosa.* **A)** Whole-cell extracts of *P. aeruginosa* strain PAO1 were incubated in either PBS or pronase (100 µg/mL) for 2 hours, resolved by SDS-PAGE together with purified PAO1 LPS, transferred to a membrane, and probed with TTR. TTR was detected using a specific anti-TTR antibody. Lane assignments were as follows: Lanes 1 and 4; whole-cell extracts incubated in PBS, lanes 2 and 5; whole-cell extracts treated with pronase, lanes 3 and 6 purified PAO1 LPS. Lanes 1–3 were probed with TTR, whereas lanes 4–6 were processed in parallel without TTR probing as control. Molecular weight markers (kDa) are indicated on the left. **B)** *P. aeruginosa* PAO1 cells were pretreated for 15 minutes at 37°C with pronase (100 μg/mL), proteinase K (100 μg/mL), or PBS (control), then washed and used to coat microtiter plate wells. Whole cell ELISA-based binding assays showing the interaction of recombinant human TTR with treated bacterial cells. Bound TTR was detected using a specific polyclonal anti-TTR antibody. Data represent three independent experiments performed in duplicate; error bars indicate standard deviation.

To identify the *P. aeruginosa* component(s) responsible for mediating the interaction with TTR, whole-cell extracts of strain PAO1 were resolved by polyacrylamide gel electrophoresis, transferred to a membrane, and probed with TTR. A distinct low–molecular-weight band was detected in the membrane incubated with TTR but was absent from the control membrane not exposed to TTR (Fig 2A). Notably, this band persisted even after protease treatment of the extract, suggesting that the TTR-reactive species might represent a lipopolysaccharide (LPS)-associated component rather than a protein. Consistent with this hypothesis, purified PAO1 LPS yielded a band identical in size and appearance to that observed in the whole-cell extracts.

To further evaluate whether surface-exposed proteins contribute to TTR binding, we examined TTR association with *P. aeruginosa* cells pretreated with proteinase K or pronase. Protease pretreatment did not affect TTR binding (Fig 2B), indicating that surface proteins are not major determinants of the interaction in either strain. Protease activity on the whole cell extracts or the intact bacterial cells was validated by quantifying the loss of the surface-associated protein EF-Tu [29]. As expected, protease treatment markedly reduced the presence of EF-Tu in whole-cell extracts (S1A Fig) and substantially decreased anti–EF-Tu antibody binding to entire bacterial cells (S1B Fig), confirming efficient degradation of both total and surface-exposed proteins.

**Fig 3:**
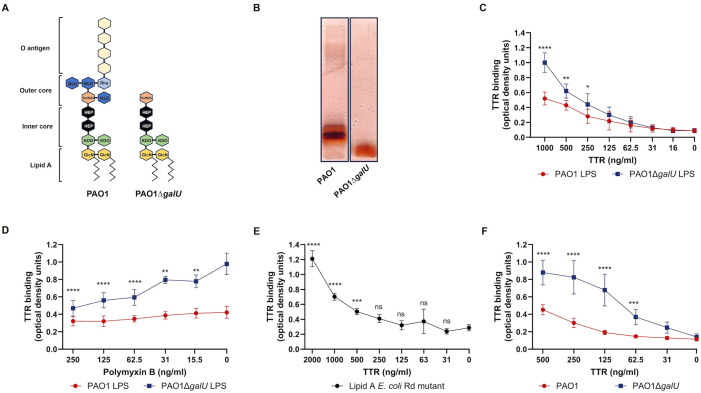
Lipopolysaccharide lipid A binding to TTR. **A)** Schematic representation of the LPS structure from *P. aeruginosa* wild-type strain PAO1 and its isogenic mutant PAO1Δ*galU*. Monosaccharides are represented as hexagons in different colors. Abbreviations: GlcN (Glucosamine), KDO (D-manno-oct-2-ulosonic acid), HEP (Heptose), GLC (Glucose), Rha (Rhamnose), GalNAla (Galactosamine). **B)** Silver-stained gel showing LPS profiles from PAO1 and PAO1Δ*galU*. **C)** Binding of human recombinant TTR to purified LPS from PAO1 and PAO1Δ*galU*, assessed by ELISA. LPS-coated wells were incubated with TTR, and bound protein was detected using a specific polyclonal antibody. **D)** Competitive binding assay showing the effect of decreasing concentrations of polymyxin B on TTR binding to LPS from PAO1 and PAO1Δ*galU*. Assays were performed as described in panel **C. E)** Binding of human recombinant TTR to purified lipid A from *E. coli* (Rd mutant) assessed by ELISA. Lipid A-coated wells were incubated with TTR, and bound protein was detected using a specific polyclonal antibody. **F)** Binding of recombinant TTR to whole cells of PAO1 and PAO1Δ*galU*, measured by whole-cell ELISA. Data points represent the mean of at least three independent experiments performed in duplicate. Error bars indicate standard deviation. In panel D, each concentration of polymyxin B was compared with the control condition (TTR binding in the absence of the inhibitory component, polymyxin **B)**. In panel E, the absorbance values corresponding to TTR binding to lipid A were compared with those obtained in the absence of TTR. Statistical analyses for panels D and E were performed using two-way ANOVA followed by Dunnett’s multiple-comparison test. *p* < 0.05; *p* < 0.01; *p* < 0.001; *p* < 0.0001.

To corroborate the ligand blot findings, we investigated the binding of recombinant human TTR to immobilized LPS. For these experiments, we used LPS isolated from the wild-type strain PAO1 and the isogenic *galU*-deficient mutant (PAO1Δ*galU*). Interruption of *galU* gene results in production of LPS devoid of O antigen (rough LPS) and truncated LPS core (Fig 3A and B). The results showed that TTR binds dose-dependently to LPS (Fig 3C). The complete LPS from the wild-type strain PAO1 showed a lower degree of TTR binding compared with the truncated LPS from the *galU*-deficient mutant (Fig 3C). Based on the LPS structure of PAO1Δ*galU*, our findings suggest that lipid A constitutes the binding site for TTR within the LPS molecule. To validated that TTR directly interacts with lipid A, we performed TTR binding experiments to LPS in the presence of polymyxin B. This lipid A binding compound inhibited binding of TTR to the LPS from PAO1Δ*galU* in a dose-dependent manner, reaching significance at all concentrations tested (Fig 3D). Polymyxin B slightly inhibited the binding of TTR to the LPS of PAO1 without reaching statistical significance. Furthermore, TTR binding assays using purified lipid A confirmed that TTR interacts with this region of the LPS (Fig 3E).

In accordance with the results obtained with purified LPS, TTR bound more efficiently to the *galU*-deficient mutant lacking the O-antigen and the complete core structure of LPS, but still exposing lipid A on its surface, than to the parent strain PAO1 (Fig 3F). Given the LPS structure of PAO1Δ*galU,* these results suggest that lipid A is a major TTR binding partner on the surface of *P. aeruginosa* and that this interaction occurs in an O antigen–dependent manner.

### TTR agglutinates and reduces the viability of *P. aeruginosa*

**Fig 4:**
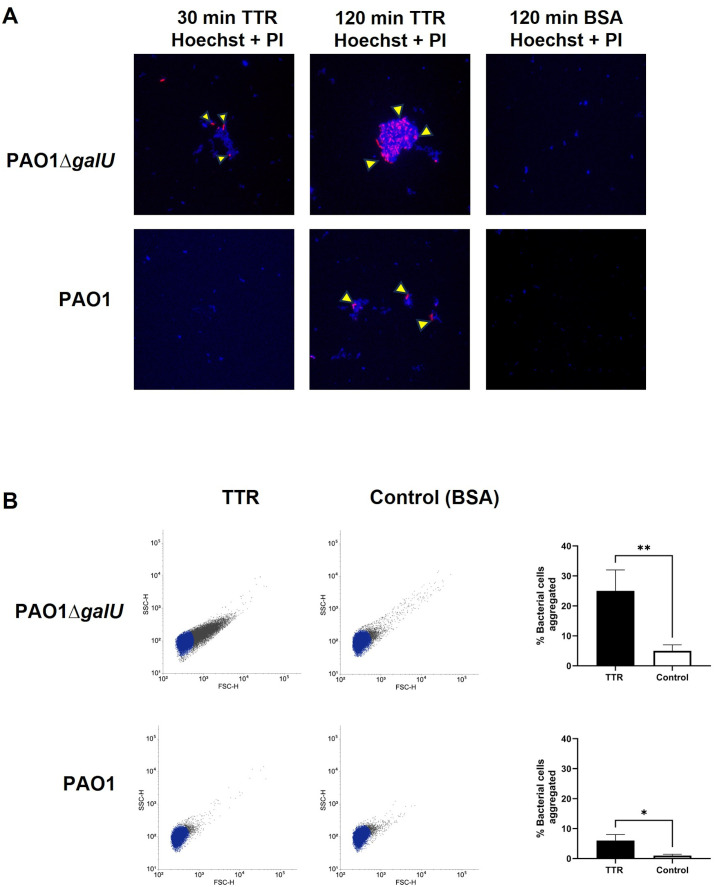
TTR induces agglutination of *P. aeruginosa.* **A)** Confocal microscopy of TTR-induced bacterial aggregates. *P. aeruginosa* wild-type strain PAO1 and its isogenic *galU*-deficient mutant (PAO1Δ*galU*) were incubated with either recombinant human TTR or BSA (both 5 μM in PBS). Bacterial cells were stained with Hoechst (blue, marking all cells) and PI (red, marking dead cells). Images were acquired at 30 and 120 minutes. Yellow arrows indicate PI-positive (dead) cells. **B)** Flow cytometry analysis of TTR-induced agglutination. Samples from panel A (120 min) were analyzed using a FACSverse cytometer. Forward scatter (FSC-H) reflects cell size, while side scatter (SSC-H) indicates internal complexity. Agglutinated bacteria (black dots) exhibit increased FSC and SSC signals. Shown plots and images are representative of three independent experiments. Quantification of aggregate formation is shown on the right. Data represent the mean ± SD of three independent experiments performed in duplicate. Statistical analysis was performed using a two-tailed *t*-test. *p < 0.05 ** *p* < 0.01.

Transthyretin (TTR) is classified as an amyloidogenic protein, as it possesses an intrinsic tendency to misfold and self-assemble under specific conditions, forming insoluble aggregates and tissue deposits known as amyloids [30,31]. It has been suggested that membrane surfaces, depending on their chemical composition, can serve as catalytic sites that promote the aggregation of amyloidogenic proteins [32,33]. To investigate whether the binding of TTR to *P. aeruginosa* induces the formation of aggregates, we studied the interaction of the bacteria with purified TTR using confocal microscopy. We utilized Hoechst and PI to monitor both cell agglutination and cell death, respectively, over time. We observed the formation of bacterial aggregates within just 30 minutes of incubating PAO1Δ*galU* with TTR at the physiological concentration of the human serum (5 μM) (Fig 4A). These aggregates became even more clearly visible after 2 hours of incubation. TTR also agglutinated the parent strain PAO1, but its effects were less pronounced compared to PAO1Δ*galU*, which correlates with the differential binding to TTR to these strains (Fig 3F). Bovine serum albumin (BSA), used as a control, had no effect on the aggregation of *P. aeruginosa*. These findings were further supported by flow cytometry analysis, which quantified the percentage of bacterial cells aggregated by TTR. The results demonstrated that TTR, but not BSA, significantly agglutinated both PAO1Δ*galU* and wild-type PAO1 strains (Fig 4B). Furthermore, pre-incubation of TTR with specific anti-TTR antibodies inhibited the formation of *P. aeruginosa* PAO1Δ*galU* aggregates by 45% ± 8.9 (*p* < 0.004) showing that aggregation is mediated by the interaction of TTR with the bacteria. These observations were extended to two clinical isolates of *P. aeruginosa*, B75 and B60 (S2 Fig). TTR mediated agglutination of strain B75, but it had no effect on B60.

**Fig 5:**
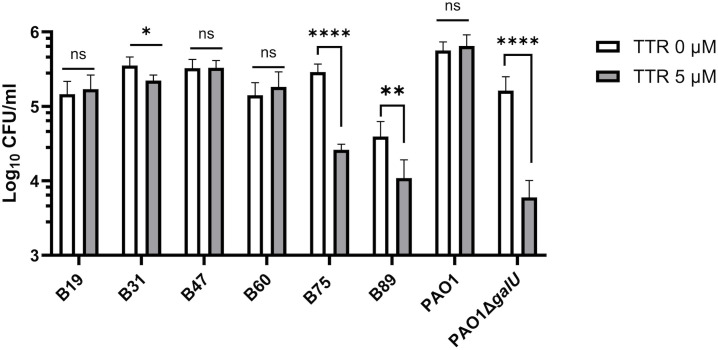
Antimicrobial activity of TTR. The survival of *P. aeruginosa* clinical isolates from bloodstream infections, along with the wild-type strain PAO1 and its isogenic *galU*-deficient mutant PAO1Δ*galU*, was evaluated following a 2-hour incubation with human recombinant TTR at 5 μM in PBS. Data are presented as the mean of at least three independent experiments performed in duplicate, with error bars representing the standard deviation. Statistical analysis comparing each strain to the untreated control was performed using a two-tailed *t*-test. Significance levels were denoted as follows: * *p* < 0.05; **p < 0·01; **** *p* < 0·0001.

Interestingly, we observed the presence of dead bacteria within the aggregates (indicated with yellow arrows in Fig 4A) suggesting that bacterial agglutination precedes a decrease in bacterial viability. In this context, some evidence suggests that amyloid proteins may have antimicrobial activity [32–35]. To quantitatively assess whether TTR has antimicrobial activity, we measured the survival of several *P. aeruginosa* clinical isolates incubated with TTR at the physiological concentration in human serum (5 μM). We observed significant variability in the survival rates of different *P. aeruginosa* strains following treatment with TTR (Fig 5). Strains that showed a high degree of aggregation induced by TTR—such as B75 or the *galU*-deficient mutant PAO1Δ*galU*—exhibited a marked reduction in viability nearly to 90%. In contrast, strains with a low degree of agglutination, such as PAO1 and B60, showed no reduction in viability. These results indicate that TTR promotes agglutination, a process that correlates with decreases in the viability of some *P. aeruginosa* strains.

### *P. aeruginosa* binds to the N-terminal region of TTR which contains a sequence with antimicrobial activity

**Fig 6:**
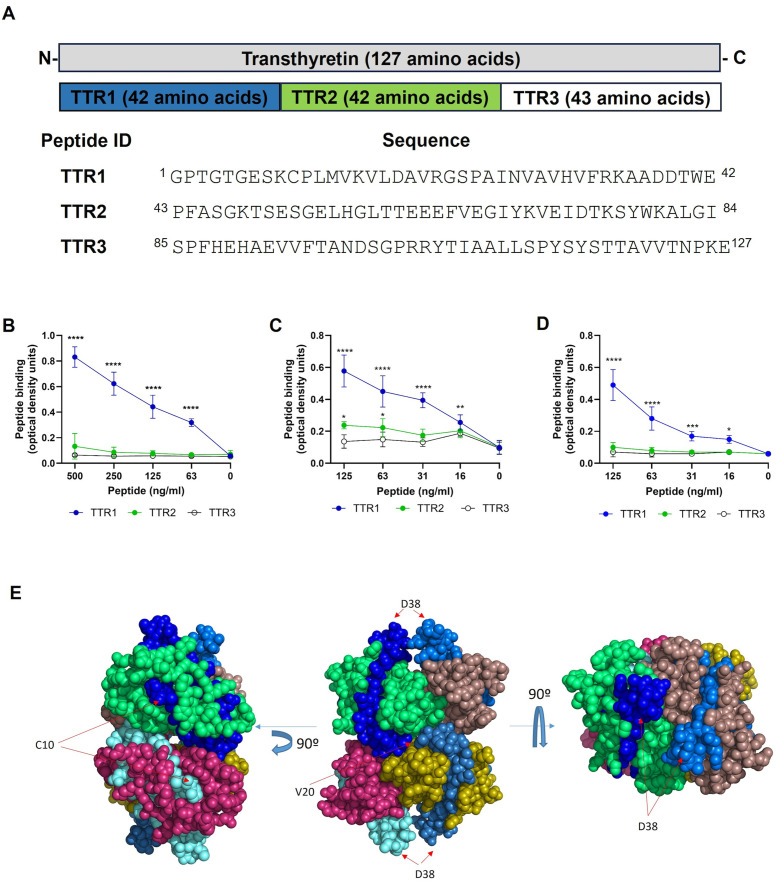
Identification of the *P. aeruginosa* binding site in TTR. **A)** Location in EF-TU and sequence of the synthetic peptides (Peptides ID; TTR1, TTR2 and TTR3) covering the TTR protein sequence (upper grey box, excluding the signal peptide) (UniProt P02766), that were used to identify the LPS binding site are shown. **B, C** and **D** ELISA binding assay demonstrating the interaction between the biotinylated synthetic peptides derived from TTR and the lipid A from *E. coli* (Rd mutant) **(B)**, the LPS from PAO1Δ*galU* **(C)** and whole cells of PAO1Δ*galU* **(C)**. **E)** The crystal structure of the TTR tetramer (Protein Data Bank code 4N85) is shown from three perspectives. Each monomer within the tetramer is distinguished by a different color. The amino acid residues corresponding to the TTR1 peptide are highlighted in varying shades of blue on each monomer. The crystal structure images were generated using PyMOL Molecular Graphics System Version 1.3, Schrödinger LLC. In panels **B, C** and **D**, data points represent the mean of at least three independent experiments performed in duplicate. Error bars indicate standard deviation. Statistical significance was determined by two-way ANOVA with Dunnett’s multiple comparison test. Comparisons were made between each data point and the corresponding control values without peptide. * *p* < 0·05; **p < 0·01; *** *p* < 0·001; ****p < 0·0001.

To gain insight into the mechanism by which TTR reduces the viability of *P. aeruginosa*, we aimed to identify the lipid A-binding region within TTR. To this end, we used a library of synthetic N-terminal biotinylated peptides spanning the entire amino acid sequence of a single TTR monomer (excluding the signal peptide) (UniProt P02766), which comprises 127 residues (Fig 6A). Microtiter plate wells coated with lipid A from *E. coli* (Rd mutant), LPS or whole cells from the mutant PAO1Δ*galU* were incubated with decreasing concentrations of each peptide. Quantitative ELISA analysis demonstrated that only peptide TTR1, representing the first 42 amino acid residues of the N-terminus, exhibited a dose-dependent interaction either with the lipid A or the LPS and the bacterial cells from PAO1Δ*galU* (Fig 6B, 6C and 6D, respectively). The 3-dimensional structure of TTR described by Yokoyama et al [36], reveals that this region is exposed on the surface of the molecule (Fig 6E).

Interestingly, a detailed computational analysis of the N-terminal region of TTR, which binds to the bacteria, based on its net charge, hydrophobicity, and length, revealed the presence of an amino acid sequence with potential antimicrobial activity [37]. Some studies have highlighted the striking resemblance between the membrane-disrupting mechanisms of antimicrobial peptides and those observed in amyloid peptides and proteins [33]. In fact, some amyloid peptides are now being considered as potential antimicrobial agents [32–34]. We hypothesized that either the whole TTR molecule or a peptide derived from its N-terminal region could kill *P. aeruginosa* by disrupting the bacterial envelope. To test this hypothesis, we synthesized a peptide derived from TTR1, designated TTR1′, and evaluated its bactericidal activity against *P. aeruginosa*. Given that TTR consists of four identical monomers (Fig 6E), each containing the TTR1’ peptide, we tested four concentrations corresponding to peptides derived from one (5 µM), two (10 µM), three (15 µM), or four (20 µM) TTR monomers, reflecting physiological serum levels. As a control, we used a peptide derived from the central region of TTR, designated TTR2′, which, based on our earlier findings, does not interact with the bacteria.

**Fig 7:**
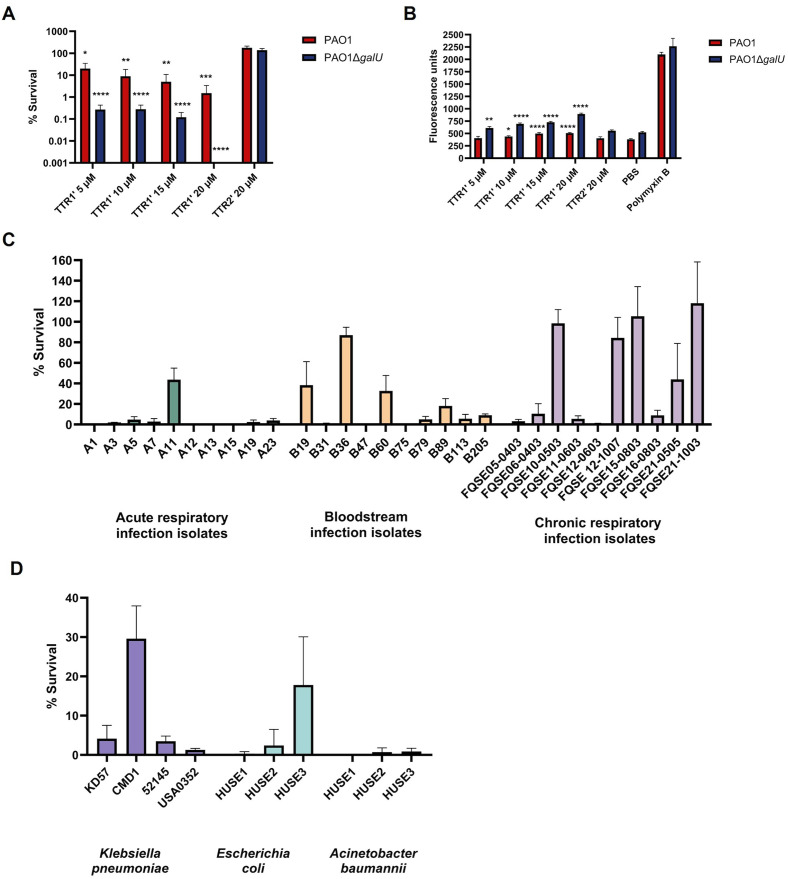
Antimicrobial activity of a human transthyretin-derived peptide. **(A)** *P. aeruginosa* wild-type strain PAO1 and its isogenic *galU*-deficient mutant (PAO1Δ*galU*) were incubated for 2 hours at 37°C with increasing concentrations of the peptide TTR1’ (derived from the N-terminal region of human TTR), TTR2’ (derived from the central region of TTR), or PBS (control). Bacterial viability was assessed by plating and expressed as a percentage relative to PBS-treated controls (100% survival). **(B)** Outer membrane permeability was evaluated by measuring NPN fluorescence after 10-minute incubation of PAO1 and PAO1Δ*galU* strains with increasing concentrations of TTR1’, TTR2’, or PBS. **(C** and **D)** Survival of *P. aeruginosa*, *K. pneumoniae*, *E. coli* and *A. baumannii* clinical isolates was assessed after 2-hour incubation with TTR1’ (20 μM) at 37°C. Viability was expressed relative to PBS-treated controls. All results represent the mean ± standard deviation from at least three independent experiments performed in duplicate. Statistical significance was assessed using a two-tailed t-test, comparing each group with the 100% survival observed in the control (panel A), and by one-way ANOVA with Dunnett’s multiple comparison test, comparing each group with PBS (panel B). * *p* < 0·05, ** *p* < 0·01, *** *p* < 0·001, **** *p* < 0·0001.

TTR1’ reduced the viability of *P. aeruginosa* PAO1Δ*galU* at all tested concentrations. At 20 µM, TTR1’ reduced the viability of this strain by more than 5 logs. In contrast, the reference strain PAO1 was less susceptible to TTR1’ than PAO1Δ*galU*, although a concentration of 20 µM reduced its viability by almost 99% (Fig 7A). Conversely, the peptide TTR2’ had no effect on the viability of either strain, suggesting that the antimicrobial activity of TTR relies on the N-terminal region of the molecule.

To determine whether the peptide TTR1’ was reducing *P. aeruginosa* viability by disrupting membrane integrity, we assessed membrane permeability using the hydrophobic fluorescent probe N-Phenyl-1-naphthylamine (NPN). NPN exhibits increased fluorescence upon entering permeabilized bacterial membranes. Treatment with TTR1’ led to a dose-dependent increase in NPN fluorescence, indicating enhanced membrane permeability in both strains, PAO1 and PAO1Δ*galU*, although in agreement with survival experiments, the effect of TTR1’ was more pronounced in PAO1Δ*galU*. In contrast, the control peptide TTR2’ had no effect (Fig 7B).

To extend our observations to *P. aeruginosa* clinical isolates, we evaluated the effect of TTR1′ on thirty additional isolates obtained from diverse sources, including acute respiratory infections, bloodstream infections and chronic respiratory infections. As shown in Fig 7C, TTR1′ reduced viability by more than 90% in nearly 70% of the isolates tested, while only 13.33% of the isolates were fully resistant to the peptide. Given that lipid A—the target of TTR1’—is a highly conserved molecular motif among Gram-negative bacteria, we next examined the effect of TTR1’ on the viability of several clinical isolates of *K. pneumoniae*, *E. coli*, and *A. baumannii*. Treatment with TTR1’ reduced the viability of most of the tested isolates by approximately 2 logs (corresponding to ~90% killing; Fig 7D). Overall, these findings indicate that TTR, or a peptide derived from its N-terminal region, binds to lipid A and exhibits broad antimicrobial activity against Gram-negative bacteria mediated by interacting with and disrupting the bacterial membrane.

## Discussion

In this work, we employed a proteomic approach to obtain a comprehensive profile of human serum proteins that interact with *P. aeruginosa* and to identify novel host factors that could illuminate host–pathogen interactions, using *P. aeruginosa* as a model organism. Our analysis identified multiple proteins previously reported to be involved in the pathogenesis of *P. aeruginosa* or other bacterial pathogens, supporting the robustness of the approach. In addition, highly abundant serum proteins such as albumin were not detected (table S1), indicating effective discrimination against nonspecific binding. Notably, we identified several proteins—including ceruloplasmin, coagulation factor V and TTR—that have not previously been implicated in interactions with *P. aeruginosa* or other bacterial pathogens and therefore merit further investigation. We acknowledge several limitations of our proteomic approach. First, the analysis was performed using a single *P. aeruginosa* strain (PAO1), which may not capture the phenotypic diversity of this species. Second, bacterial surface–associated proteins were eluted using a high–salt buffer, a condition that likely favors the recovery of proteins bound through ionic interactions; proteins associated via other mechanisms, such as covalent linkages, may therefore have been underrepresented. Third, human serum was supplemented with EDTA to prevent complement activation, which may have altered the binding profile by inhibiting or promoting the association of some proteins. These limitations may have influenced our ability to detect certain proteins that interact with *P. aeruginosa* but were not identified in this study.

Among the previously unrecognized proteins that interact with bacteria, TTR emerged as particularly noteworthy, as it has not previously been implicated as a host-derived factor in antimicrobial defense or bacterial pathogenesis. TTR is a plasma transport protein that primarily carries thyroxine and retinol, the latter via complex formation with retinol binding protein. Under physiological conditions, TTR assembles into a soluble, stable homotetramer [38,39]. However, membrane surfaces have been shown to facilitate the aggregation of amyloidogenic proteins, such as TTR. In turn, such proteins can disrupt the structural integrity of cellular membranes [32–34]. Based on this evidence, we hypothesized that TTR–bacterial membrane interactions may also promote the formation of agglutinated complexes containing both TTR and bacterial cells. Collectively, our results show that all clinical isolates of *P. aeruginosa* tested bind TTR, and that TTR rapidly agglutinates these bacteria via direct interaction with lipid A, the innermost region of the LPS molecule. Given that lipid A—the TTR target—is highly conserved among Gram-negative bacteria, this phenomenon may be broadly relevant and non-specific, a characteristic typical of innate immune effectors.

The TTR-binding experiments with the parent strain PAO1 and its isogenic *galU*-deficient mutant demonstrate that TTR binding is influenced by the presence of the LPS O-antigen, which—similar to other innate effectors such as complement [40]—can confer resistance to TTR-mediated killing. Consequently, TTR’s agglutination and bactericidal activity depend on LPS O antigen, which may explain why certain isolates are resistant to TTR-mediated killing.

We demonstrate that TTR binding to *P. aeruginosa* occurs through the N-terminal region of the host protein, which is prominently exposed on the homotetramer. Although some residues within this region are also involved in binding the natural ligands of TTR—such as thyroxine, which engages Lys15, and retinol-binding protein, which interacts with Lys15 and Leu17—these interactions are unlikely to interfere with bacterial binding. This is because <1% of circulating TTR is bound to thyroxine at any given time [41], while only 20–60% is associated with retinol-binding protein [42]. Consistent with this, our TTR-binding assays using human serum demonstrate robust TTR binding to *P. aeruginosa* despite the presence of endogenous thyroxine and retinol-binding protein. Importantly, this N-terminal region does not contain most of the residues commonly mutated in TTR amyloidosis [43], making it unlikely that pathogenic TTR variants would confer susceptibility to *P. aeruginosa* infection.

Computational analysis of the TTR N-terminal sequence predicted antimicrobial activity, which we confirmed experimentally in bacterial survival assays. Amyloid-forming proteins share structural and functional features with antimicrobial peptides (AMPs): both can adopt amphipathic conformations with hydrophobic and cationic domains, preferentially bind negatively charged membranes, and require a threshold local concentration to form oligomeric, membrane-permeabilizing species. Upon reaching this threshold, oligomers assemble into transmembrane pores via proposed structural models [34]. This likely explains why the N-terminal peptide showed greater antimicrobial activity than full-length TTR, aided by improved access to membrane targets due to its small size compared to the entire protein. In fact, most *P. aeruginosa* clinical isolates were highly susceptible to the TTR derived AMP. Interestingly, 40% of the isolates from chronic infections were resistant to the TTR derived AMP. Although the number of tested isolates is insufficient to draw definitive conclusions, it is plausible that the capsule—a known AMP resistance factor [44], often overexpressed in the mucoid phenotype common among these isolates [45]—may mediate this resistance.

The biological relevance of this finding hinges on *in vivo* generation of such peptides. Interestingly, TTR can be cleaved by the protease plasmin between Lys48 and Thr49, generating the 49–127 fragment previously implicated in amyloid deposition [46,47]. Notably, the TTR-derived antimicrobial peptide identified in our study (residues 8–35) nearly corresponds to the complementary fragment produced by this cleavage event. Moreover, this peptide has been detected in human blood [48], although its physiological function has not yet been defined. Intriguingly, our findings suggest a dual role for plasmin activity during infection: while *P. aeruginosa* exploits plasmin to promote tissue dissemination [19], plasmin may also cleave host proteins such as TTR, generating cryptic AMPs that contribute to innate defense. This represents a novel host strategy wherein proteolytic enzymes, co-opted by the pathogen for virulence, simultaneously generate antimicrobial effectors from abundant plasma proteins. Thus, plasmin acts as a molecular fulcrum in the host–pathogen interaction, mediating both bacterial virulence and host defense, underscoring a sophisticated biochemical tug-of-war that may influence infection outcomes.

Notably, TTR is also present in other fluids such as CSF or vaginal fluid, which lacks most innate immune effectors present in blood. In fact, Patgaonkar et al. identified TTR-derived AMPs in the rabbit vaginal fluid [49]. We propose that TTR and its cryptic peptide derivatives may contribute to innate host defense, with a particularly important antimicrobial function in the central nervous system and other susceptible sites, including the urinary and respiratory tracts.

We have not yet performed *in vivo* experiments using TTR knockout mice to directly test whether TTR is required for host defense against *P. aeruginosa*. Nevertheless, clinical observations provide indirect support for such a role: patients with TTR amyloidosis receiving TTR gene–silencing therapies—who exhibit profoundly reduced circulating TTR levels—experience a higher incidence of urinary and respiratory tract infections [50]. Although redundancy across innate immune pathways likely mitigates overt vulnerability, these findings raise the possibility that TTR deficiency contributes to impaired antimicrobial defense, highlighting the need for further investigation.

We propose that during infection, TTR binds to Gram-negative bacteria via lipid A, providing a catalytic surface that promotes local TTR aggregation and formation of protein–bacteria complexes, as has been described for other amyloid peptides with antimicrobial activity, including the Alzheimer’s disease-associated amyloid β-protein [51] among others [34]. This process may represent an early step in AMP generation by host proteases or by microbial proteases. Together, our findings suggest that TTR contributes to host defense both through direct bacterial binding and lysis, and via proteolytic release of cryptic AMPs. We propose that this mechanism reflects an ancestral innate immune strategy, which gram-negative bacteria have partially evaded through LPS modifications, but which may still operate under specific infection conditions.

## Materials and methods

### Bacterial strains

*P. aeruginosa* reference strain PAO1 and its isogenic *galU*-deficient mutant (PAO1Δ*galU*), were previously described [52]. The interruption of *galU* gene results in production of lipopolysaccharide (LPS) devoid of O antigen (rough LPS) and truncated LPS core. This study also included three sets of clinical *P. aeruginosa* isolates, each comprising ten strains. The first set was obtained from epidemiologically unrelated patients from the intensive care unit with acute respiratory infections [29]. The second set belongs to larger collection of isolates from chronic respiratory infections [53]. Finally, the third set of strains was collected from patients with bloodstream infections [8].

Clinical isolates from *Klebsiella pneumoniae* (5 isolates), *Escherichia coli* (3 isolates) and *Acinetobacter baumannii* (3 isolates) were also included in this study. Bacterial cells were grown in Luria Bertani (LB) broth at 37°C with shaking or in LB solidified with 1.5% agar.

### Human serum and recombinant human transthyretin

Human serum was purchased from Sigma-Aldrich, Merck and stored at −80°C until its use*.* In some experiments, sera were heat inactivated at 56°C for 30 min prior to use or supplemented with 5 mM EDTA to inhibit complement activation.

Human recombinant TTR was generously provided by Dr. Mizuguchi from Toyama University, Toyama, Japan, who purified the protein as previously described [54]. The purity of the protein (>97%) was confirmed by sodium dodecyl sulfate-polyacrylamide gel electrophoresis (SDS-PAGE) analysis.

### Proteomic analysis of human serum proteins bound to *P. aeruginosa*

We employed a proteomic approach to identify human serum proteins that interact with *P. aeruginosa*. A pooled human serum sample, supplemented with 5 mM EDTA to inhibit complement activation, was mixed at a final concentration of 20% with approximately 10¹⁰ bacterial cells of the *P. aeruginosa* reference strain PAO1, resuspended in 200 µL of phosphate-buffered saline (PBS). The mixture was incubated for 1 hour at 37°C to allow protein binding. Following incubation, the bacteria were washed six times with PBS to remove unbound proteins, ensuring stringent conditions. Surface-bound proteins were then eluted using 200 µL of 2 M NaCl. Both the final wash and elution fractions were subjected to enzymatic digestion and analyzed by mass spectrometry, following a previously described protocol [55]. Subsequent proteomic data analysis was conducted as outlined in the same reference [55]. To ensure specificity and reproducibility, only proteins that consistently showed a relative abundance in the eluted fraction at least twofold higher than in the final wash across five independent experiments were considered.

### LPS purification

LPSs from strain PAO1 and from its isogenic *galU*-deficient mutant (PAO1Δ*galU*) were isolated by the phenol-water method as previously described [56]. These LPSs were analyzed by SDS-PAGE using 15% polyacrylamide gels and the separated bands were visualized by silver staining [56]. Lipid A from *E. coli* (Rd mutant) was purchased from Sigma-Aldrich, Merck.

### Ligand blot assays

*P. aeruginosa* PAO1 cells (1 ×  10⁹) were resuspended in 50 µL of PBS alone or PBS containing pronase (final concentration, 100 µg/ml) and incubated for 2 h at 37°C. Following incubation, 50 µL of loading buffer was added, and bacterial suspensions were boiled for 5 min. Samples were resolved by SDS-PAGE and transferred onto a PVDF membrane.

Membranes were blocked for 1 h at room temperature with PBS containing 1% (w/v) BSA and then incubated for 1 h at room temperature with transthyretin (TTR; 2 µg/ml) diluted in PBS or with PBS alone as a negative control. After extensive washing with PBS, membrane-bound TTR was detected using a rabbit polyclonal anti-TTR antibody (Sigma-Aldrich, Merck) diluted 1:5,000 in PBS. Membranes were subsequently incubated with an alkaline phosphatase–conjugated goat anti-rabbit IgG secondary antibody (Sigma-Aldrich, Merck) diluted 1:5,000 in PBS. Signal detection was performed using the Fast 5-bromo-4-chloro-3-indolyl phosphate/nitroblue tetrazolium (BCIP/NBT) substrate kit (Sigma-Aldrich, St. Louis, MO).

### TTR binding assays

The binding of TTR to bacterial cells was determined by ELISA. Briefly, 96-well round-bottom polystyrene microtiter plates were coated overnight at 37ºC with 10^8^ bacterial cells resuspended in PBS. In some experiments, bacterial cells were pretreated (15 minutes, 37°C) with pronase E (100 μg/mL; Sigma-Aldrich, Merck), proteinase K (100 μg/mL; Sigma-Aldrich, Merck), or buffer and washed 3 times with PBS before coating the plates. Next, wells were blocked with PBS containing 1% Bovine Serum Albumin (BSA) and incubated with TTR at different concentrations, or heat-inactivated human sera (10%) diluted in PBS. Wells incubated with PBS instead of TTR or human sera were used as controls. TTR was detected with a specific rabbit polyclonal antibody (Sigma-Aldrich, Merck) diluted 1:5000 in PBS. Finally, wells were incubated with an alkaline phosphatase-conjugated goat anti-rabbit immunoglobulin G (Sigma-Aldrich, Merck) diluted 1:5000 in PBS, and developed with p-nitrophenyl phosphate (Sigma-Aldrich, Merck) in 50 mM carbonate-bicarbonate buffer, pH 9.6, 5 mM MgCl_2_. Absorbance was measured at 415 nm. Optical densities from negative control wells incubated with PBS instead of TTR were always below 0.1 and were subtracted from the experimental values. Washing steps with PBS were included between incubations with antibodies that were performed for 1 h at 37ºC.

Binding of TTR to LPS was also determined by ELISA using the protocol described above but instead using microtiter plate wells that were coated overnight at 4ºC with 1 µg of purified LPS dissolved in 100 µl of 50 mM bicarbonate (pH 9.6) or 2 h at 37ºC with 50 ng of lipid A dissolved in 50 µl of ethanol. In addition, binding of TTR to immobilized LPS was measured in the presence of increasing concentrations of polymyxin B sulfate (Sigma-Aldrich, Merck), or buffer. All binding experiments were independently repeated at least three times, with technical duplicates included in each run.

### Confocal microscopy

*P. aeruginosa* cells (10^7^ Colony Forming Units (CFU)) grown at 37ºC in LB to the early exponential growth phase (OD_600_ = 0.3) were washed and resuspended in PBS and incubated at different time points with TTR at the physiologic concentration in human serum (5 μM) [9] in PBS, pH 7.4. BSA was used as a negative control. In some experiments, TTR was pre-incubated for 1h at 37ºC with a specific anti-TTR rabbit polyclonal antibody (Sigma-Aldrich, Merck) diluted 1/2000 in PBS before the incubation with the bacterial cells. An aliquot of the bacterial suspension was stained with Hoechst (Sigma-Aldrich, Merck) and propidium iodide (PI) (Sigma-Aldrich, Merck) and imaged using a laser scanning confocal microscope (Leika TCS-SPE). Hoechst was excited using a 405-nm argon laser, with emission collected between 450–500 nm, while PI was excited using 532-nm diode laser, with emission collected between 580–700 nm.

### Fluorescence-Assisted Cell Sorting assays

A bacterial suspension was prepared and incubated with TTR as described for the confocal microscopy experiments. After incubation, an aliquot of the bacterial suspension was stained with PI. Bacterial cells were subjected to Fluorescence-Assisted Cell Sorting (FACS) analysis using a FACSverse cytometer (BD Biosciences) and a dot-plot was generated by representing the low angle forward scattering (FSC-H) in the x-axis and the side scattering (SSC-H) in the y-axis to analyze the size and complexity of the cell cultures. Results were analyzed using BD FACSsuite software.

### Bacterial viability assays

Approximately 1 × 10^5^ CFU of *P. aeruginosa*, grown to exponential phase at 37°C in LB medium, were incubated at 37°C with either full-length TTR (TTR; 5 μM, corresponding to the physiological human serum concentration), the N-terminal-derived peptide TTR1′ (sequence: SKCPLMVKVLDAVRGSPAINVAVHVFRK), or the central region-derived peptide TTR2′ (sequence: LHGLTTEEEFVEGIYKVEIDTKSYWKAL), each diluted in PBS at various concentrations. PBS alone served as a negative control. After 2 hours of incubation, bacterial survival was assessed by plating serial dilutions on LB agar and enumerating CFUs. Survival experiments were independently repeated at least three times, with technical duplicates included in each run.

### Identification of the *P. aeruginosa* binding site in TTR

Microtiter ELISA wells were coated with whole bacterial cells or LPS as described above and blocked with PBS-BSA. Next were incubated with decreasing concentrations of each of the three synthetic N-terminal biotinylated peptides (TTR1 (42-mer), TTR2 (42-mer) and TTR3 (43-mer) that were generated by Proteogenix. Together, these peptides span the entire human TTR sequence, excluding the signal peptide (see Fig 6A). After three washing steps, peptides were detected using a streptavidin alkaline phosphatase-conjugated conjugate molecule and developed with p-nitrophenyl phosphate (Sigma-Aldrich, Merck) in 50 mM carbonate-bicarbonate buffer, pH 9.6, 5 mM MgCl_2_. Absorbance was measured at 415 nm. Background signal was determined for wells without any peptide. Background values were < 0.1 optical density units. Incubations with streptavidin alkaline phosphatase-conjugated conjugate molecule was made at 37°C for 1 hour and washing steps with PBS were included after each incubation. All samples were measured in duplicates and the data are presented as averaged values from at least three independent assays.

### Membrane permeability assay

Disruption of the outer membrane was determined by using the N-phenyl-1-napthylamine (NPN) (Sigma-Aldrich, Merck) uptake assay, as previously described [57]. Briefly, *P. aeruginosa* cultures in the exponential growth phase (OD₆₀₀ = 0.3) were harvested, washed, and resuspended in PBS. Bacterial suspensions were then incubated at 37°C for 10 min with NPN (final concentration: 10 μM) in the presence of either TTR1’ (5–20 μM), TTR2’ (20 μM), polymyxin B (1 μg/mL) (Sigma-Aldrich, Merck), or PBS (vehicle control). Fluorescence intensity was measured using a microplate reader with an excitation wavelength of 350 nm and an emission wavelength of 420 nm, indicating NPN uptake as a proxy for membrane permeability. All samples were measured in duplicates and the data are presented as averaged values from at least three independent assays.

### Statistical analysis

Statistical analyses and graphical representations were performed using GraphPad Prism (version 10.6.0). Quantitative variables were analyzed using appropriate statistical tests, each specified in the corresponding Fig legend. Data represent results from at least three independent experiments.

## Supporting information

S1 Table List of human serum proteins bound to *P. aeruginosa* PAO1. (DOCX)

S1 Fig Protease activity on whole-cell extracts and intact *P. aeruginosa* cells. Protease activity was validated by assessing degradation of the abundant bacterial protein elongation factor Tu (EF-Tu) in whole-cell extracts (A) or loss of surface-associated EF-Tu on intact bacterial cells (B). (A) Whole-cell extracts of *P. aeruginosa* strain PAO1 were incubated for 2 h in PBS or with pronase (100 µg/mL), resolved by SDS–PAGE, and transferred to a membrane. EF-Tu was detected using an anti-EF-Tu monoclonal antibody (mAb H900, Hycult Biotech). (B) Intact *P. aeruginosa* cells were pretreated for 15 min at 37°C with pronase (100 µg/mL), proteinase K (100 µg/mL), or PBS (control), washed, and used to coat microtiter plate wells. Surface-associated EF-Tu was quantified by whole-cell ELISA using the anti-EF-Tu monoclonal antibody (mAb H900, Hycult Biotech), serving as a control for protease activity. Data represent three independent experiments performed in duplicate; error bars indicate standard deviation. Statistical significance was determined by one-way ANOVA followed by Tukey’s multiple-comparisons test. **** p < 0.0001.
(TIFF)

S2 Fig TTR induces agglutination of *P. aeruginosa* clinical isolates. A) Confocal microscopy of TTR-induced bacterial aggregates. *P. aeruginosa* clinical isolates B75 and B60 were incubated with either recombinant human TTR or BSA (both at 5 μM in PBS). Bacterial cells were stained with Hoechst (blue, marking all cells) and PI (red, marking dead cells). Images were acquired at 30 and 120 minutes. Yellow arrows indicate PI-positive (dead) cells. B) Flow cytometry analysis of TTR-induced agglutination. Samples from panel A (120 min) were analyzed using a FACSverse cytometer and aggregate formation was quantified. Data represent the mean ± SD of three independent experiments performed in duplicate. Statistical analysis was performed using a two-tailed *t*-test. ** *p* < 0·01.
(TIFF)
