# Supplementary figures and images for: Transthyretin is a novel innate immune effector against Gram negative bacteria
Source: PLoS Pathog. 2026 Mar 23;22(3):e1014086. doi: 10.1371/journal.ppat.1014086 (PMC13035231; doi:10.1371/journal.ppat.1014086)

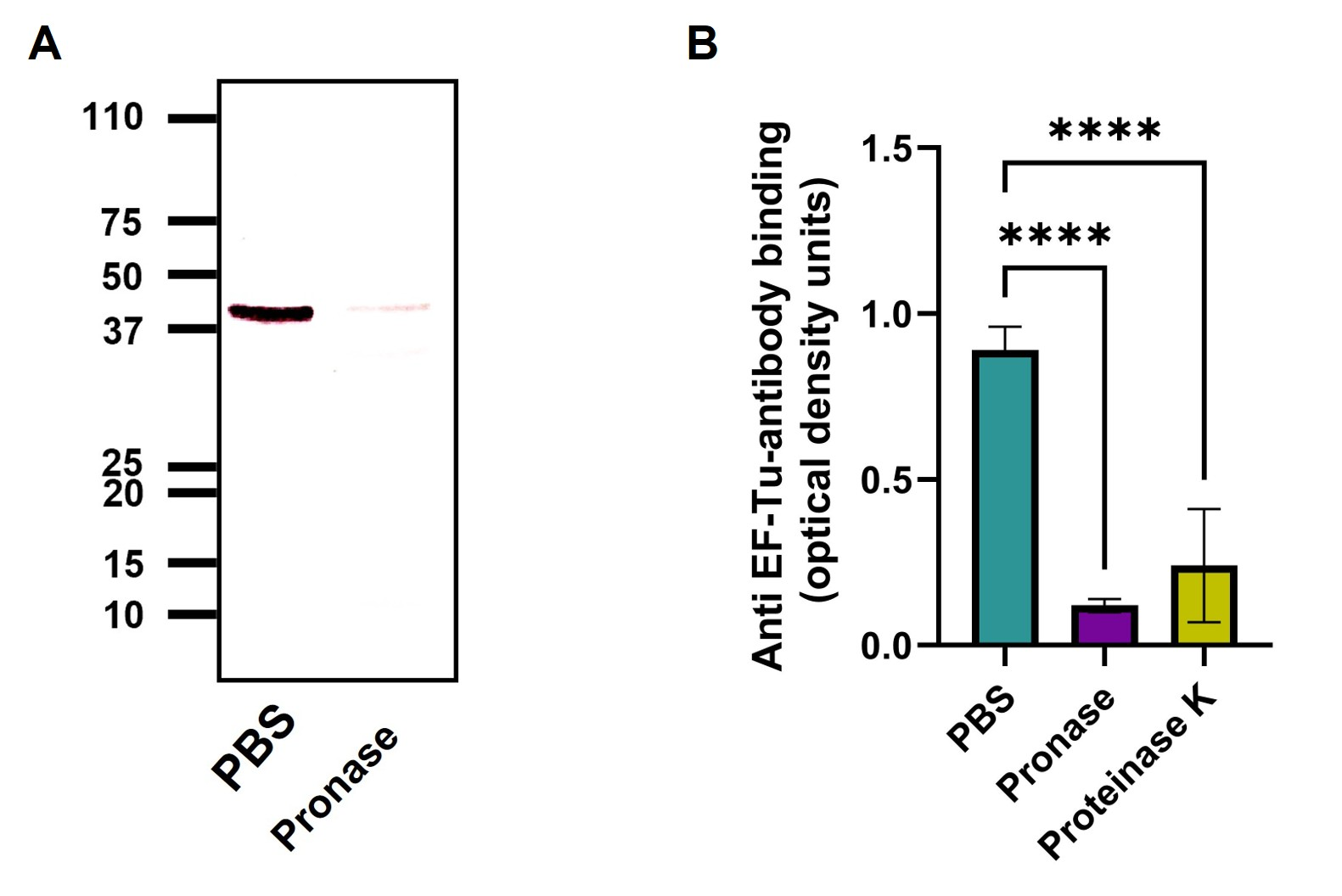

Supplement: S1 Fig — Protease activity was validated by assessing degradation of the abundant bacterial protein elongation factor Tu (EF-Tu) in whole-cell extracts (A) or loss of surface-associated EF-Tu on intact bacterial cells (B). (A) Whole-cell extracts of P. aeruginosa strain PAO1 were incubated for 2 h in PBS or with pronase (100 µg/mL), resolved by SDS–PAGE, and transferred to a membrane. EF-Tu was detected using an anti-EF-Tu monoclonal antibody (mAb H900, Hycult Biotech). (B) Intact P. aeruginosa cells were pretreated for 15 min at 37°C with pronase (100 µg/mL), proteinase K (100 µg/mL), or PBS (control), washed, and used to coat microtiter plate wells. Surface-associated EF-Tu was quantified by whole-cell ELISA using the anti-EF-Tu monoclonal antibody (mAb H900, Hycult Biotech), serving as a control for protease activity. Data represent three independent experiments performed in duplicate; error bars indicate standard deviation. Statistical significance was determined by one-way ANOVA followed by Tukey’s multiple-comparisons test. **** p < 0.0001. (TIFF) [file ppat.1014086.s002.tiff]

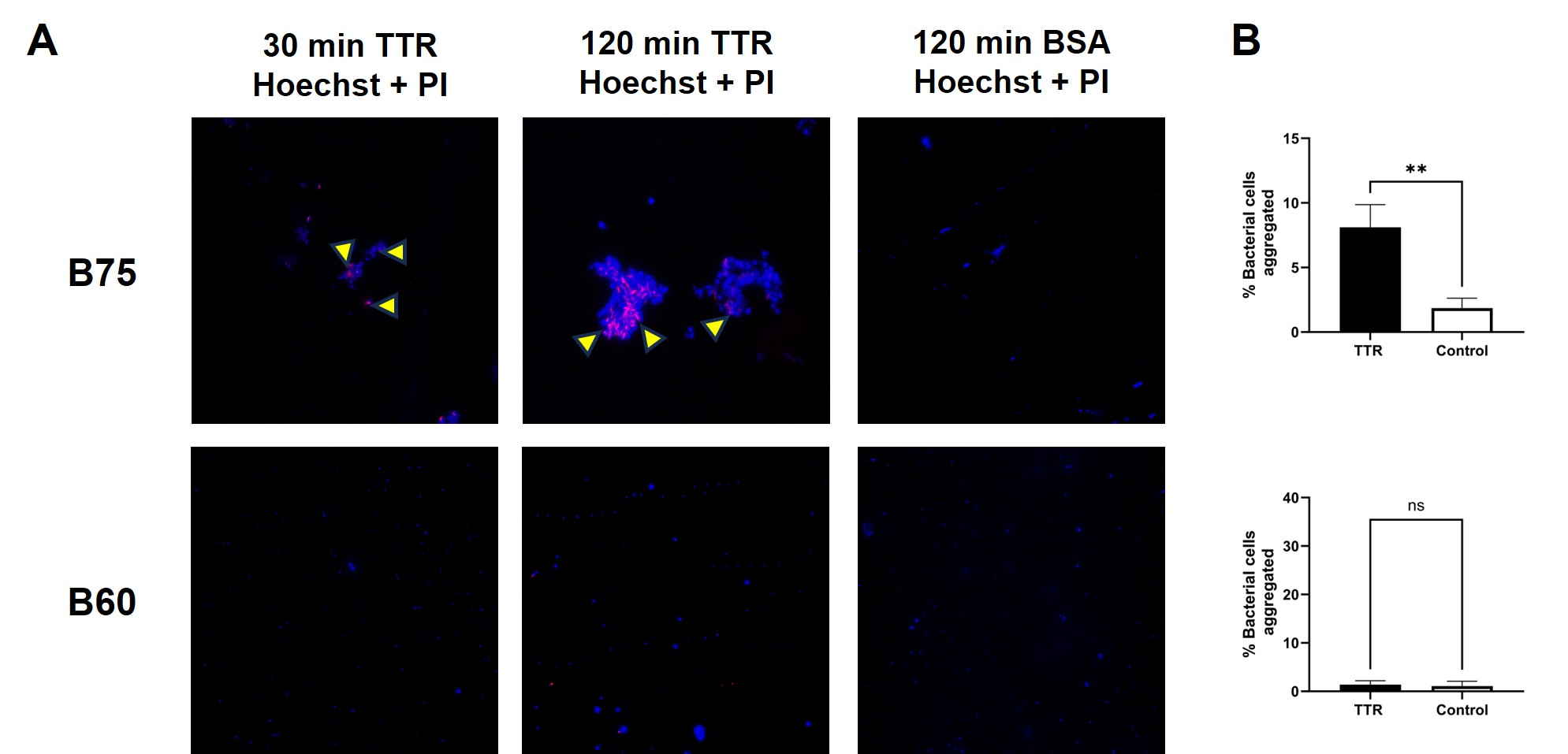

Supplement: S2 Fig — A) Confocal microscopy of TTR-induced bacterial aggregates. P. aeruginosa clinical isolates B75 and B60 were incubated with either recombinant human TTR or BSA (both at 5 μM in PBS). Bacterial cells were stained with Hoechst (blue, marking all cells) and PI (red, marking dead cells). Images were acquired at 30 and 120 minutes. Yellow arrows indicate PI-positive (dead) cells. B) Flow cytometry analysis of TTR-induced agglutination. Samples from panel A (120 min) were analyzed using a FACSverse cytometer and aggregate formation was quantified. Data represent the mean ± SD of three independent experiments performed in duplicate. Statistical analysis was performed using a two-tailed t-test. ** p < 0·01. (TIFF) [file ppat.1014086.s003.tiff]
