# Supplementary material for: Transthyretin is a novel innate immune effector against Gram negative bacteria
Source: PLoS Pathog. 2026 Mar 23;22(3):e1014086. doi: 10.1371/journal.ppat.1014086 (PMC13035231; doi:10.1371/journal.ppat.1014086)
Supplement: Supplementary file 2 [file ppat.1014086.s001.docx]

Table S1. List of human serum proteins bound to *P. aeruginosa* PAO1

Accession	Description	Coverage [%]	Peptides	Unique Peptides	Molecular weight [kDa]	Calculated isoelectric point	Abundance Ratio (Elute/Last Wash) (a)	P value (a)
P06727	Apolipoprotein A-IV OS=Homo sapiens OX=9606 GN=APOA4 PE=1 SV=3	40	15	15	45.4	5.38	5.2594	3.26919E-05
P04114	Apolipoprotein B-100 OS=Homo sapiens OX=9606 GN=APOB PE=1 SV=2	20	65	65	515.3	7.05	23.3398	1E-15
P04003	C4b-binding protein alpha chain OS=Homo sapiens OX=9606 GN=C4BPA PE=1 SV=2	58	28	28	67	7.3	6.7072	1.07739E-08
P20851	C4b-binding protein beta chain OS=Homo sapiens OX=9606 GN=C4BPB PE=1 SV=1	33	6	6	28.3	5.14	15.6504	0.175145284
O43866	CD5 antigen-like OS=Homo sapiens OX=9606 GN=CD5L PE=1 SV=1	46	9	9	38.1	5.47	12.3726	1.81455E-13
P00450	Ceruloplasmin OS=Homo sapiens OX=9606 GN=CP PE=1 SV=1	32	22	22	122.1	5.72	4.317	0.036896758
P10909	Clusterin OS=Homo sapiens OX=9606 GN=CLU PE=1 SV=1	28	11	11	52.5	6.27	4.4658	0.000997582
P12259	Coagulation factor V OS=Homo sapiens OX=9606 GN=F5 PE=1 SV=4	4	7	7	251.5	6.05	11.965	3.23419E-11
P02745	Complement C1q subcomponent subunit A OS=Homo sapiens OX=9606 GN=C1QA PE=1 SV=2	40	8	8	26	9.11	14.0644	8.0621E-11
P02746	Complement C1q subcomponent subunit B OS=Homo sapiens OX=9606 GN=C1QB PE=1 SV=3	43	8	8	26.7	8.63	16.603	1E-15
P02747	Complement C1q subcomponent subunit C OS=Homo sapiens OX=9606 GN=C1QC PE=1 SV=3	37	8	8	25.8	8.41	16.3086	5.68961E-05
P00736	Complement C1r subcomponent OS=Homo sapiens OX=9606 GN=C1R PE=1 SV=2	20	8	8	80.1	6.21	3.6104	0.007484296
P09871	Complement C1s subcomponent OS=Homo sapiens OX=9606 GN=C1S PE=1 SV=1	18	8	8	76.6	4.96	6.5174	0.020866565
P08603	Complement factor H OS=Homo sapiens OX=9606 GN=CFH PE=1 SV=4	23	20	18	139	6.61	3.8196	0.012486995
Q03591	Complement factor H-related protein 1 OS=Homo sapiens OX=9606 GN=CFHR1 PE=1 SV=2	17	5	3	37.6	7.39	4.247	0.020860558
P02671	Fibrinogen alpha chain OS=Homo sapiens OX=9606 GN=FGA PE=1 SV=2	4	2	2	94.9	6.01	6.8232	0.008647698
Q15485	Ficolin-2 OS=Homo sapiens OX=9606 GN=FCN2 PE=1 SV=2	33	5	5	34	6.77	30.2742	0.002086483
P00739	Haptoglobin-related protein OS=Homo sapiens OX=9606 GN=HPR PE=2 SV=2	52	15	5	39	7.09	15.5568	1.4658E-09
P04196	Histidine-rich glycoprotein OS=Homo sapiens OX=9606 GN=HRG PE=1 SV=1	10	5	5	59.5	7.5	10.4596	0.015286217
P19827	Inter-alpha-trypsin inhibitor heavy chain H1 OS=Homo sapiens OX=9606 GN=ITIH1 PE=1 SV=3	7	5	5	101.3	6.79	2.6672	0.023995848
P19823	Inter-alpha-trypsin inhibitor heavy chain H2 OS=Homo sapiens OX=9606 GN=ITIH2 PE=1 SV=2	14	9	9	106.4	6.86	17.9708	0.206030355
Q14624	Inter-alpha-trypsin inhibitor heavy chain H4 OS=Homo sapiens OX=9606 GN=ITIH4 PE=1 SV=4	13	9	9	103.3	6.98	4.141	0.013962848
P01042	Kininogen-1 OS=Homo sapiens OX=9606 GN=KNG1 PE=1 SV=2	28	10	10	71.9	6.81	18.2644	2.45213E-09
P05164	Myeloperoxidase OS=Homo sapiens OX=9606 GN=MPO PE=1 SV=1	8	4	4	83.8	8.97	6.9014	0.016178131
P00747	Plasminogen OS=Homo sapiens OX=9606 GN=PLG PE=1 SV=2	57	37	37	90.5	7.24	9.1946	1E-15
P00734	Prothrombin OS=Homo sapiens OX=9606 GN=F2 PE=1 SV=2	22	10	10	70	5.9	6.7106	2.4744E-09
P35542	Serum amyloid A-4 protein OS=Homo sapiens OX=9606 GN=SAA4 PE=1 SV=2	20	2	2	14.7	9.07	6.643	0.022468134
P02766	Transthyretin OS=Homo sapiens OX=9606 GN=TTR PE=1 SV=1	69	7	7	15.9	5.76	7.3034	0.006168813
P04004	Vitronectin OS=Homo sapiens OX=9606 GN=VTN PE=1 SV=1	30	12	12	54.3	5.8	10.4384	1E-15
P02768	Serum albumin OS=Homo sapiens OX=9606 GN=ALB PE=1 SV=2	91	104	104	69.3	6.28	0.2514	1E-15

(a) Mean abundance ratio of protein bound to bacteria relative to the amount of protein detected in the final wash prior to elution, calculated from five independent experiments. *P* values indicate the statistical significance of differences between the eluted fraction and the final wash.
